# Transcription factor regulatory modules provide the molecular mechanisms for functional redundancy observed among transcription factors in yeast

**DOI:** 10.1186/s12859-019-3212-8

**Published:** 2019-12-27

**Authors:** Tzu-Hsien Yang

**Affiliations:** Department of Information Management, National University of Kaohsiung, 700, Kaohsiung University Rd, Kaohsiung, 81148 Taiwan

**Keywords:** Transcription regulation, TF regulatory module, TF functional redundancy

## Abstract

**Background:**

Current technologies for understanding the transcriptional reprogramming in cells include the transcription factor (TF) chromatin immunoprecipitation (ChIP) experiments and the TF knockout experiments. The ChIP experiments show the binding targets of TFs against which the antibody directs while the knockout techniques find the regulatory gene targets of the knocked-out TFs. However, it was shown that these two complementary results contain few common targets. Researchers have used the concept of TF functional redundancy to explain the low overlap between these two techniques. But the detailed molecular mechanisms behind TF functional redundancy remain unknown. Without knowing the possible molecular mechanisms, it is hard for biologists to fully unravel the cause of TF functional redundancy.

**Results:**

To mine out the molecular mechanisms, a novel algorithm to extract TF regulatory modules that help explain the observed TF functional redundancy effect was devised and proposed in this research. The method first searched for candidate TF sets from the TF binding data. Then based on these candidate sets the method utilized the modified Steiner Tree construction algorithm to construct the possible TF regulatory modules from protein-protein interaction data and finally filtered out the noise-induced results by using confidence tests. The mined-out regulatory modules were shown to correlate to the concept of functional redundancy and provided testable hypotheses of the molecular mechanisms behind functional redundancy. And the biological significance of the mined-out results was demonstrated in three different biological aspects: ontology enrichment, protein interaction prevalence and expression coherence. About 23.5% of the mined-out TF regulatory modules were literature-verified. Finally, the biological applicability of the proposed method was shown in one detailed example of a verified TF regulatory module for pheromone response and filamentous growth in yeast.

**Conclusion:**

In this research, a novel method that mined out the potential TF regulatory modules which elucidate the functional redundancy observed among TFs is proposed. The extracted TF regulatory modules not only correlate the molecular mechanisms to the observed functional redundancy among TFs, but also show biological significance in inferring TF functional binding target genes. The results provide testable hypotheses for biologists to further design subsequent research and experiments.

## Introduction

Cells usually respond to environmental and physiological stress by reorganizing their DNA transcription programs, leading to correct spatial and temporal expression of different genes [1–3]. To subtly control the DNA transcription programs, transcription factors (TFs) coordinately bind to the promoter regions of their target genes and regulate the expression of these genes [4]. The precise regulation of the binding of TFs depends on the interaction of different TFs, regulatory proteins and the epigenetic materials such as nucleosome and histone modifications [5–7]. Hence understanding the roles and mechanisms of TFs in transcriptional regulation is an important task and remains the on-going research in systems biology and molecular biology.

Current genome-wide experimental methods for understanding the behaviors of how TFs control cellular gene expression can be divided into two categories [8, 9]. The first type of experiment is based on the chromatin immunoprecipitation (ChIP) techniques. Using the antibodies designed to recognize specific TFs followed by tiling arrays or next generation sequencing methods, the binding target genes of these specific TFs can be found if the identified binding sequences are mapped to the promoter regions or the proximal genic regions of the target genes [4]. Recent studies also showed that distal binding regulatory sites exist in cells and can be further analyzed in the identified ChIP binding events [10]. Whether with binding sequences in the proximal/promoter regions or in the distal regions, together the identified target genes are called the direct binding targets of the TFs under study. Overall the ChIP experiments can provide the cellular TF binding datasets. The second type of high-throughput technology is the TF knockout technique. Using the TF knockout experiments, the expression difference of genome-wide gene expression levels between the mutant-type and wild-type cell lysates generated by the knockout of certain TFs can be measured using the tiling arrays or high-throughput sequencing methods [2, 11]. These TF knockout experiments can identify the direct and indirect regulatory target genes for the knocked-out TF. Since these two techniques convey different aspects for gene transcription regulation, different researches have been conducted to try to dig out the molecular mechanisms of TF regulation in gene transcription based on integrating these two experimental data and/or other different genome-wide datasets [12, 13].

While integrating the TF binding data and the TF knockout data can help reveal some novel findings in the transcription regulation mechanisms of TFs, it is somewhat surprising that researchers have found that these two datasets overlap with each other at a very low percentage [12, 13]. This raises an interesting issue that most of the TF binding events identified by the ChIP experiments do not show significant expression level change after these TFs are knocked-out. Different statistical data analysis pipelines have been proposed for analyzing the TF binding datasets and the TF knockout datasets. Their results all led to this puzzling phenomenon and hence the results showed that experimental noises and data analysis techniques do not account for the low overlap percentage between these two experiments [2, 4, 13, 14]. Therefore, it is believed that some other possible biological explanations should be proposed for this puzzling issue.

Researchers have been trying to find possible cellular reasons for the low overlap percentage of the TF binding data and the TF knockout data. It is shown that TFs that have biological back-up functions shared with some redundant paralogs have lower agreement between the ChIP targets and the expression level changes in knockout experiments [13]. These TF’s evolutional homology relationships indicate that back-up mechanisms may exist in cells thus compensating the effects when some TFs were lost in some cellular conditions. This observation is later further explored by considering the functional redundancy of different TFs [15]. TFs with higher functional redundancy calculated based on Gene Ontology information have fewer common gene targets in the binding dataset and the knockout regulatory dataset. These results demonstrated that TFs with similar functions and evolutionarily conserved sequences/structures tend to show higher functional redundancy, resulting in the masking effect in the knockout experiments when compared with the ChIP results. While using the concepts of TF functional redundancy and evolution comparison can successfully correlate the low overlap to the functional behavior in cells, they did not provide the real molecular mechanisms behind this puzzling question. There is no easy way for biologists to further design subsequent experiments for unraveling the functional redundancy effects in cells if no such potential molecular mechanisms are extracted and proposed.

Transcription factors are thought to cooperate with one another and regulate target genes coordinately [16]. Various methods have been proposed to identify regulatory sets that extract the participating genes and TFs in certain cellular responses [17, 18]. But to elucidate the molecular mechanisms behind the functional redundancy that cause the low overlapping between the TF ChIP experiments and knockout experiments, one needs to further genome-widely consider detailed cooperative TF regulatory modules as a whole to understand the molecular basis of back-up mechanisms in transcription programs since TF regulatory modules include different interactions between the TFs and related regulatory proteins that may involve in the regulation process [19]. In yeast, researchers have tried to figure out the possible TF combinations that may involve in TF regulatory modules using the concepts of fuzzy set theory [20]. However, this only fuzzily scanned through few combinations of the transcription factor binding sites (TFBSs). This type of scanning of TF combinations did not reveal the complete TF regulatory modules, which may contain other non-TFs, non-DNA binding regulatory proteins or mediator proteins that coordinate the modular regulation. Further, this fuzzy method did not take functional redundancy into consideration and did not provide good correlation to functional redundancy. Hence in this study, we tried to devise an algorithm based on the biological knowledge of cooperativity in regulatory modules to help extract the potential TF regulatory modules and elucidate the molecular mechanisms behind the functional redundancy observed among TFs.

In this research, a novel algorithm that helps mine out the potential molecular mechanisms for the functional redundancy observed among TFs and thus elucidate the reasons of the low overlap between ChIP results and the knockout experiments is proposed. The overall method can be divided into three stages: candidate search stage, module mining stage and noise reduction stage. Based on the biological concepts that TFs may cooperate with one another thus contributing to the functional backup and redundancy phenomenon, the proposed three-staged method integrated the cellular protein-protein interaction information and the genome-wide mRNA expression data of the TF-encoding genes to mine out the potential TF regulatory modules from the TF ChIP binding dataset. The mined-out TF regulatory modules were then demonstrated that they are highly correlated to the concept of functional redundancy. Further, these TF regulatory modules provided possible molecular mechanism hypotheses behind the idea of TF functional redundancy. In addition, using these mined-out TF regulatory modules, one could identify the so-called module-inferred functional binding target genes of TFs from the original binding data. Then the biological significance of these module-inferred functional binding target gene sets was tested and the results showed that module-inferred functional binding targets are more biological significant than the original binding data. In summary, the proposed method can extract biologically significant and testable molecular mechanism hypotheses for TF functional redundancy. About 23.5% of the mined-out TF regulatory modules have been experimentally verified in the literature. In the last, a cellular example was described in detail to show some of the proven results of the mined-out pheromone response TF regulatory module.

## Results

### Overview of the algorithm

**Fig. 1:**
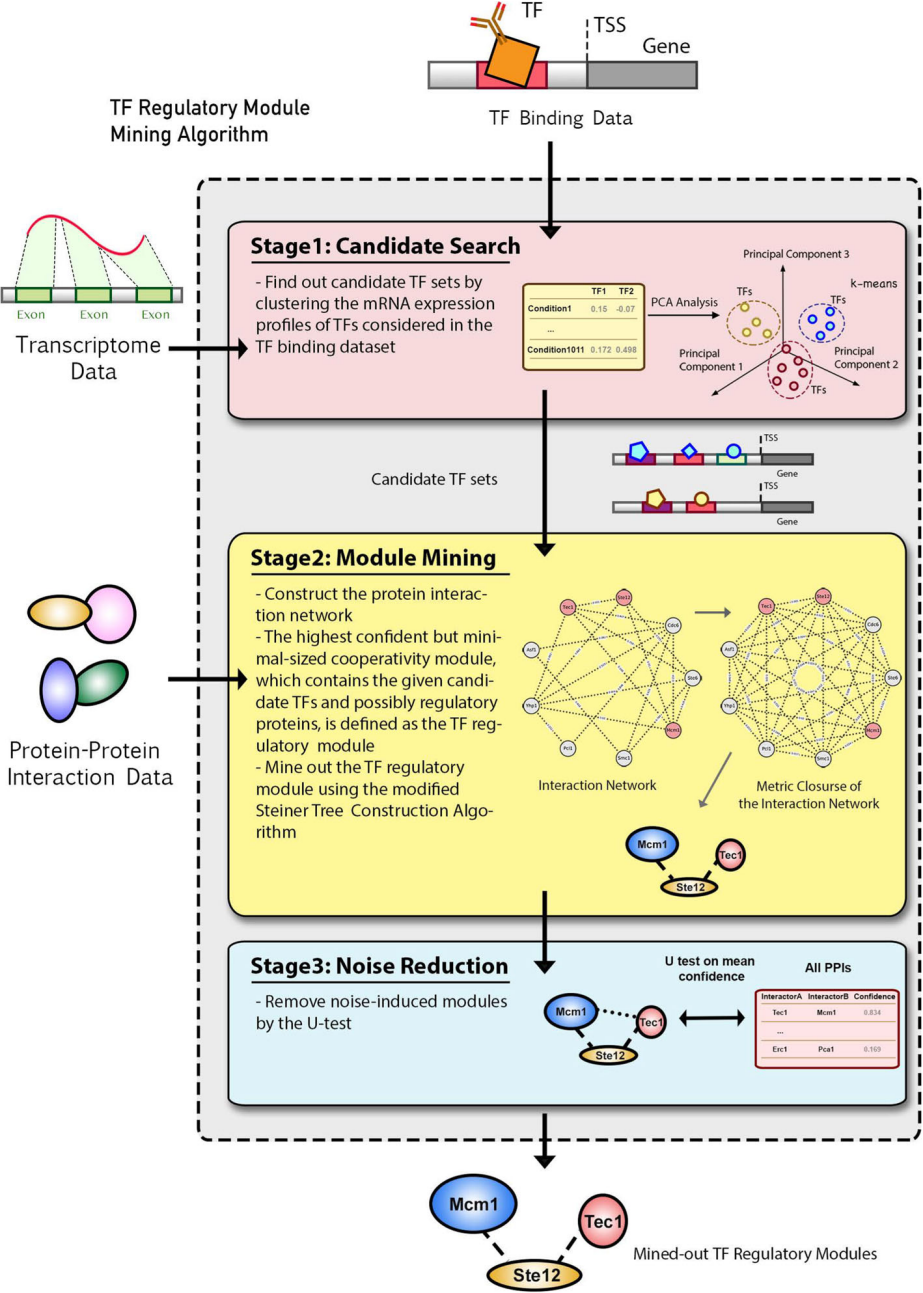
The overview of the proposed module mining algorithm. The proposed method for mining out the molecular mechanisms for functional redundancy can be divided into three stages. First, we searched for candidate TF sets from the TF binding dataset. Then based on these candidate sets, we performed the TF regulatory module mining by the modified Steiner Tree construction algorithm. In the third stage, we filtered out the noise-induced random results by confidence tests

The proposed method to mine out the potential TF regulatory modules that help elucidate the molecular mechanisms behind functional redundancy observed among TFs is summarized in Fig. 1. The algorithm can be divided into three stages. First, candidate TF sets that may be involved in the same modules were selected by integrating the binding data with the expression data adopted from the work of Ihmels et al., which consists of 1011 published expression experiments for different cellular conditions [21]. Since TFs that involve in the same regulatory module tend to have similar cellular expression, the *k*-means data clustering methods [22] was applied on the ChIP-identified binding TFs of a given gene to group co-expressed TFs into candidate TF sets. Next the protein-protein interaction network was constructed based on the data from the STRING Database [23] for extracting the molecular mechanisms of these selected candidate TF sets. A weighted network was built based on the protein-protein interaction information where the weights of the edges were defined as one minus the literature confidence provided by the STRING Database. To infer molecular mechanisms for a given candidate TF set, the module mining algorithm was devised to find the most confident but minimal-sized connected cooperative interaction sub-graph, which not only contains the candidate TFs but may also include potential non-TF regulatory proteins that are not in the given candidate TF set. In this step, the possible molecular mechanisms of TF regulatory modules were mined out. Details of the module mining algorithm can be found in the “Datasets and methods” section. Finally, to reduce the effect of noises inherited from the high-throughput technologies of expression experiments and protein-protein interaction identification experiments, the Mann-Whitney U test [24] was applied to test the confidence of the extracted modules under the hypothesis that a true TF regulatory module should have statistically higher literature confidence than the literature confidence of the whole protein-protein interaction network. The final TF regulatory modules were picked out by the algorithm based on a *p*-value threshold of 0.05 for confidence tests followed by FDR multiple hypotheses correction.

### The identified tF regulatory modules

**Fig. 2:**
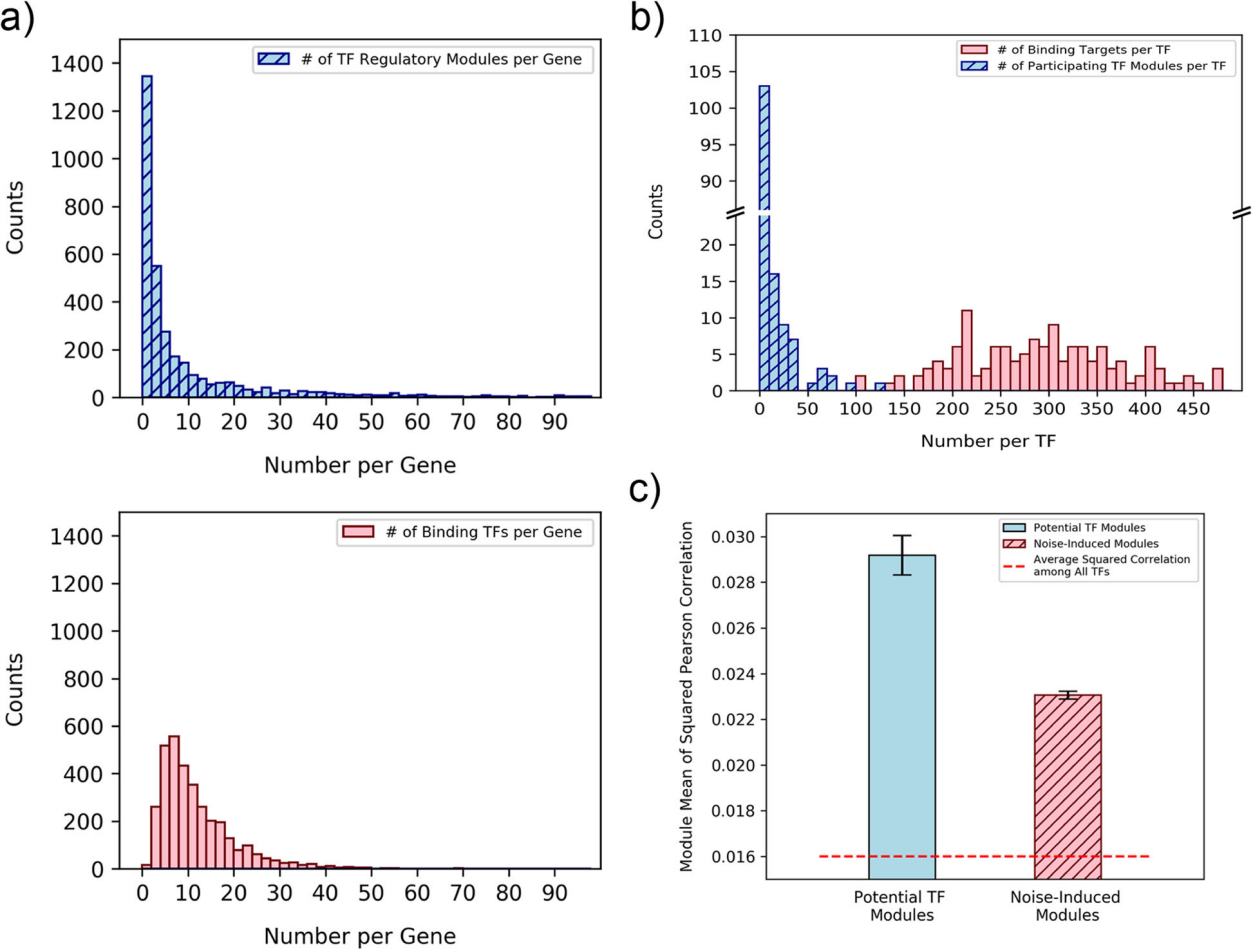
The histograms of the extracted TF regulatory modules per gene/TF and the module coherence comparison. **a** The average number of extracted TF regulatory modules per gene is smaller than the average number of binding TFs per gene. **b** The average number of participating TF regulatory modules per TF is also smaller than average the number of binding targets per TF. **c** The extracted potential TF regulatory modules showed higher coherence (average coherence = 0.029) than the coherence of the filtered-out noise-induced modules (average coherence = 0.023). The error bar in this plot indicates the standard error of the module coherence

The proposed TF module identification strategy was applied to the TF binding dataset extracted from the TF ChIP-chip experiments performed by Harbison et al. [4]. A *p*-value threshold of 0.05 was used to pick the potential TF-gene binding pairs in the TF binding dataset. To get the starting candidate sets, the mRNA expression data collected by Ihmels et al., which consists of 1,011 published expression experiments for different cellular conditions [21], was chosen to be integrated. Then the devised method constructed the protein interaction module using the interaction pairs obtained from the STRING v11 database [23]. As depicted in Fig. 1, the proposed method tried to find out all possible combinations of co-expressed TF sets in the candidate search stage. And noise-induced modules were later carefully filtered out in the noise reduction stage. After the second stage of the proposed module mining strategy (Module Mining Stage in Fig. 1), 30,588 possible TF modules were obtained. Then a multiple hypotheses-adjusted TF-module *p*-value threshold of 0.05 was adopted in the confidence tests and the method finally extracted 238 final confident TF regulatory modules that may involve in *Saccharomyces cerevisiae* transcription regulation. The distribution of the number of extracted TF regulatory modules per gene/TF was further checked and compared with the distribution of the number of TF binding sites per gene/TF (See Fig. 2a, b). As we can see in Fig. 2, the number of extracted TF regulatory modules per gene (Mean = 3) is smaller than the number of binding TFs per gene (Mean = 9). And the number of extracted participating TF regulatory modules per TF (Mean = 3) is also smaller than the number of binding targets per TF (Mean = 294). The noise-induced modules were mostly eliminated in the process. Moreover, the module coherence was defined as the average of all squared correlations between any two TFs in this module. The final extracted confident potential TF regulatory modules showed higher coherence (average coherence = 0.029) than both the average coherence between any two TFs in yeast (average coherence = 0.016) and the average coherence of the noised-induced modules (average coherence = 0.023) (Figure 2c). Hence TF co-expression was ensured in the proposed method. The details of these 238 TF regulatory modules can be found in Additional file 1. In summary, 143 of the 203 TFs studied in the Harbison TF binding dataset were shown to be involved in at least one TF regulatory module.

### TFs that involve in regulatory modules show higher functional redundancy

It has been shown that functional redundancy of transcription factors accounts for the reason why most of binding targets are not observed in the TF knock-out results [15]. And the biological mechanisms behind the concept of functional redundancy can be unraveled by mining out TF regulatory modules that may co-regulate the specified target gene. To illustrate this, the correlation of the concept of functional redundancy in TFs to the extracted TF regulatory modules was investigated. First the functional redundancy scores of TFs were calculated using the definition proposed by Wu and Lai [15] based on the Dice coefficient:

$$ FRS(t) = \max_{q} \frac{2 \mid F_{t} \cap F_{q} \mid}{ \mid F_{t} \mid + \mid F_{q} \mid}   $$

where *F*_*t*_ and *F*_*q*_ is the set of cellular functions annotated by Gene Ontology Consortium [25] for TF *t* and TF *q*, respectively. ∣*F*_*t*_∩*F*_*q*_∣ is the number of common functions annotated by GO Consortium for both *F*_*t*_ and *F*_*q*_. The functional redundancy scores are ranged between 0 and 1. The higher the score is, the more functional redundant the TF is.

**Fig. 3:**
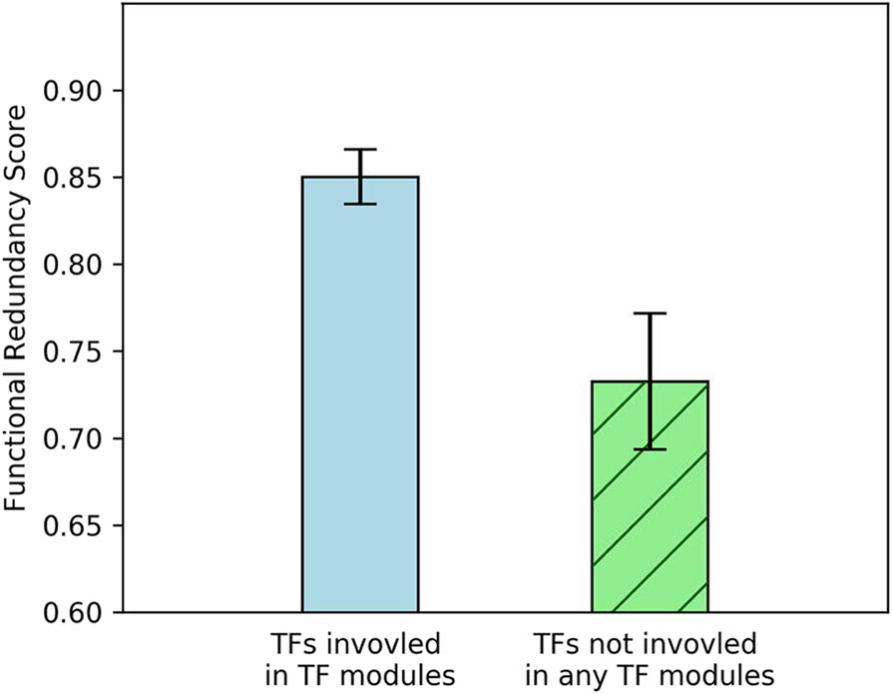
TFs categorized to function in regulatory modules showed higher functional redundancy. TFs that were shown to be involved in at least one mined-out TF regulatory module got statistically higher functional redundancy scores than TFs that were shown to be not involved in any TF modules (*p*-value = 0.0003*). The error bar in this plot indicates the standard error of the functional redundancy scores of TFs

Using the *t*-test, the functional redundancy scores of the TFs that involved in at least one TF module were compared with the functional redundancy scores of the TFs that were shown to not participate in any TF modules. The result is shown in Fig. 3. As the test results shown in the figure, the 143 TFs that were shown to be involved in at least one mined-out TF regulatory module got statistically higher functional redundancy scores (mean *FRS*=0.85) than the 61 TFs that were shown to be not involved in any TF modules (mean *FRS*=0.73) with *p*-value 0.0003*. From this analysis, it was demonstrated that TFs that involve in the mined-out TF regulatory modules are more functionally redundant than those TFs that do not cooperate in any TF modules. Hence the proposed method extracted TF regulatory modules that help explain the molecular mechanisms behind the concept of functional redundancy observed among transcription factors in yeast.

### The original TF binding data consist of 30.8% module-inferred TF-gene functional binding pairs

Since the extracted TF regulatory modules are to help biologists elucidate molecular mechanisms behind the functional redundancy observed among TFs, these modules are also supposed to be able to help identify the TF functional binding target genes from the original statistically analyzed TF binding dataset [13, 26]. Thus, one can utilized these extracted modules to assist the identification of functional TF binding target genes from the TF binding data, which were obtained from the ChIP experiments performed by Harbison et al. [4]. Based on the concept of backup-regulation mechanisms that may be involved in a TF regulatory module [2, 4, 27], a target gene of a specific TF is called a functional target if the target gene is also a knockout target or the following two conditions are satisfied for the target gene: (i) we can observe knockout evidence showing experimentally that some TF in the mined-out TF regulatory module regulates the target gene (ii) there exists another binding signal from some TF in the regulatory module to the target gene. The knockout evidence was taken from the work of Hu et al. [2], in which the cellular conditions used were the same as the conditions applied in the binding dataset. In this research, knockout-inferred functional binding pairs are the TF-gene binding signals that also demonstrate expression changes when the binding TFs are knocked-out while the pure module-inferred functional binding pairs are the TF-gene binding signals that can only be explained by using the extracted TF regulatory modules. Since the knockout-inferred functional targets can be regarded as trivial/degenerate cases in the above module-assisted functional target identification, these deduced functional binding TF-gene pairs in both cases will together be called the module-inferred functional pairs throughout this research.

**Fig. 4:**
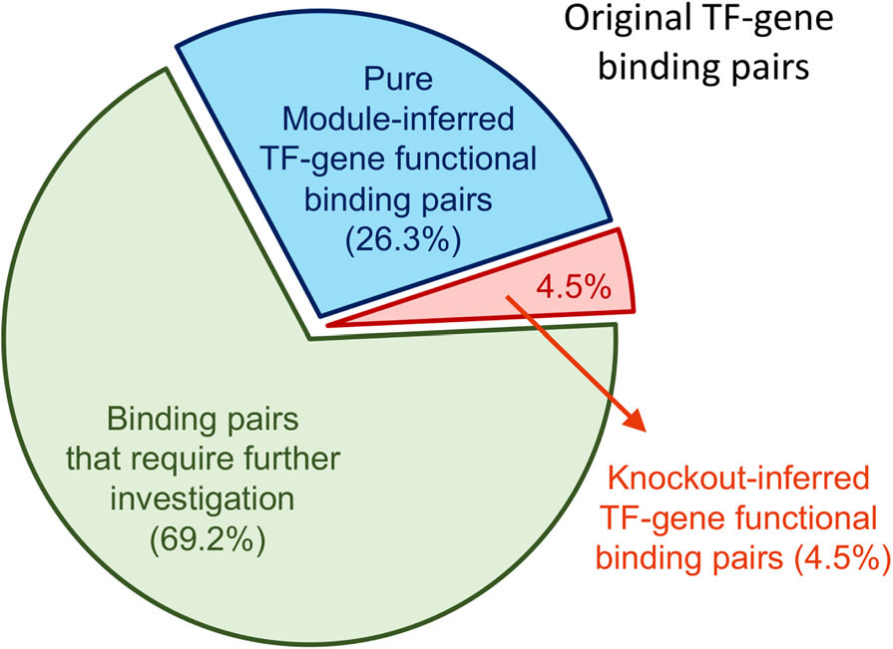
30.8% of the original TF binding data are found to be the TF module-inferred functional binding pairs. In the original Harbison ChIP dataset, 4.5% of the binding targets overlap with the TF knock-out experiments of Hu *et al*. These are called the knockout-inferred direct regulatory targets or knockout-inferred functional binding targets in this research. And utilizing the mined-out TF regulatory modules, extra 26.3% of the target genes were derived as pure module-inferred functional binding targets. In total, 30.8% of target genes are the module-inferred functional pairs

For the 203 TFs considered in the ChIP experiments of Harbison et al., 186 TFs underwent TF knockout experiments performed by Hu et al. and thus were used in inferring functional targets. After applying the proposed criteria to the original 54,218 TF-gene binding pairs of these 186 TFs, 16,713 module-inferred functional pairs for 182 out of the 186 TFs were identified (See Fig. 4). Four TFs got no functional binding targets according to the above criteria. The details of the deduced module-inferred functional binding target genes can be found in Additional file 2. Among these 16,713 module-inferred functional binding pairs (30.8% of the original binding data), only 2452 functional TF-gene pairs (4.5% of the original binding data) show expression level difference when the binding TFs are knocked-out. That is, only 4.5% of the original binding pairs, which are called knockout-inferred functional binding targets or knockout-inferred direct regulatory targets in this research, can be found to be functional using direct overlapping of the knockout data and the binding data. The low percentage of direct overlap of these two datasets were partially explained by the concept of functional redundancy observed for the involving TFs [15]. The proposed TF module identification strategy can help elucidate the biological mechanisms for TF functional redundancy, identifying extra 14,261 (26.3% of the original binding data) pure module-inferred functional TF-gene pairs (See Fig. 4).

### TF regulatory modules help deduce biological significant functional binding pairs from the original TF binding dataset

**Fig. 5:**
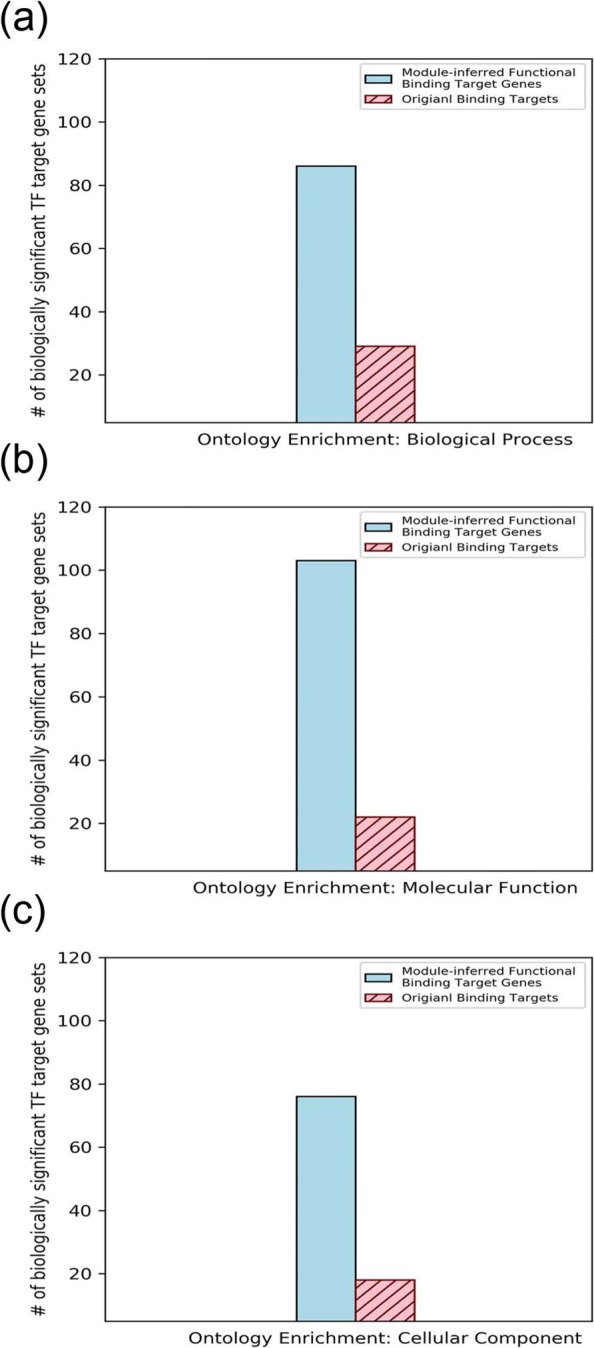
The extracted TF regulatory modules helped enrich biological significance in gene ontology. **a** For the biological process ontology, 86 (47.2%) TFs showed higher log enrichment scores in module-inferred functional binding target genes while only 29 (15.9%) TFs showed higher log enrichment scores in the original data. **b** For the molecular function ontology, 103 (56.6%) TFs showed higher log enrichment scores in module-inferred functional binding target genes while only 22 (12.1%) TFs showed higher log enrichment scores in the original data. **c** For the cellular component ontology, 76 (41.8%) TFs showed higher log enrichment scores in module-inferred functional binding target genes while only 18 (9.9%) TFs showed higher log enrichment scores in the original data

**Fig. 6:**
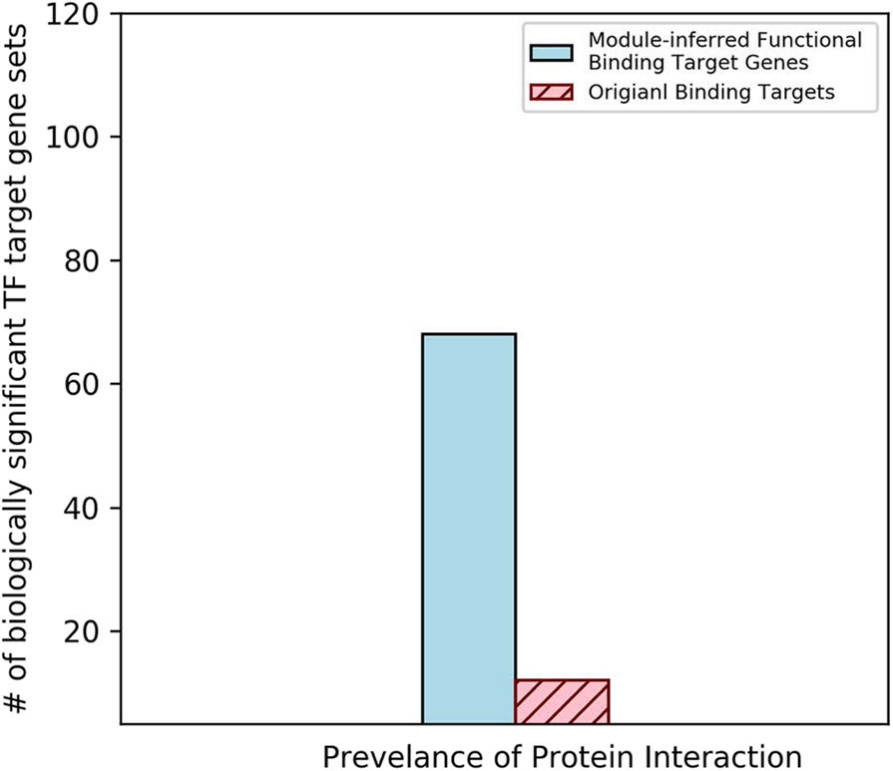
The extracted TF regulatory modules helped enrich biological significance in protein interaction prevalence. 68 (37.4%) of the module-inferred functional binding gene sets revealed prevalence of protein interaction while only 12 (6.6%) original binding gene sets did

**Fig. 7:**
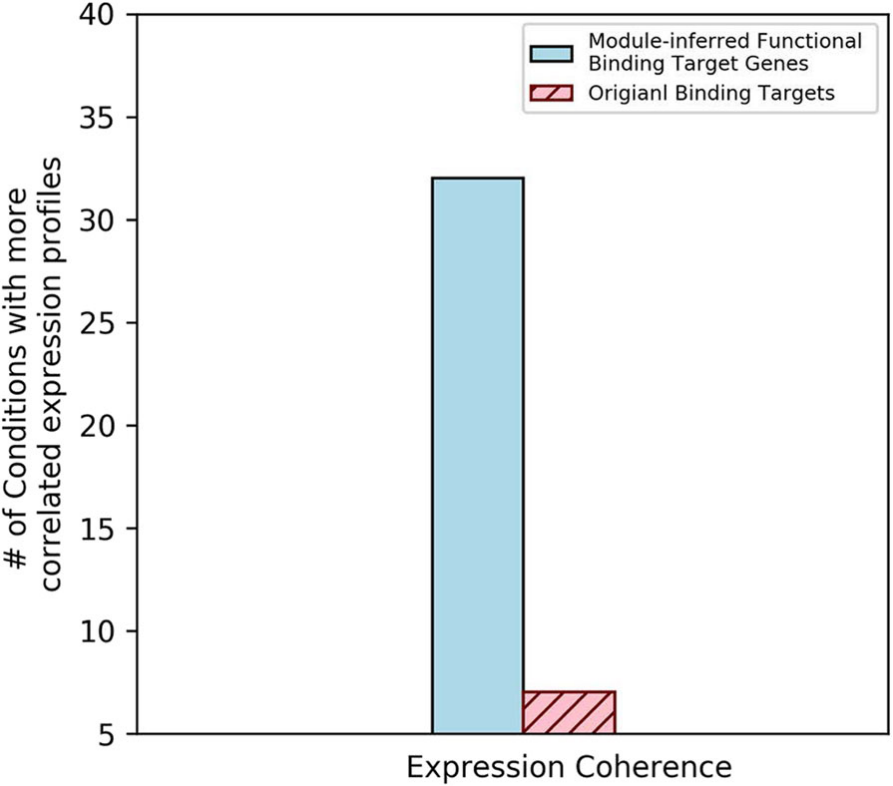
Module-inferred functional binding targets were more biologically significant in expression coherence. Module-inferred functional binding target genes had higher expression coherence in 32 different conditions while in 7 conditions the original data got higher expression coherence

To validate the biological significance of the extracted TF regulatory modules and thus the regulatory mechanisms of functional redundancy in yeast, these regulatory modules were then checked if they could help identify biologically significant module-inferred functional gene targets. The biological significance of the resulting module-inferred functional binding TF-gene pairs was demonstrated by three biological statistical tests: the TF target gene set ontology enrichment test, the test of protein interaction prevalence for the target gene sets and the expression coherence test for the target genes. The testing results are summarized in Fig. 5, 6 and 7.

#### TF module-inferred functional targets show better ontology enrichment

It is a common situation in cells that genes regulated by the same TF are usually carrying similar molecular or cellular functions [12, 26, 28]. Hence if the module-inferred TF functional targets are of importance, the module-inferred functional binding target gene sets should exhibit biological enrichment significance in their molecular and cellular ontology annotation. Using the Gene Ontology (GO) [25] information, we can characterize the ontology annotations of the target gene sets. We say that the target gene set is ontology-enriched in a GO-term if the percentage of genes in this gene set carrying this GO-term is more significantly statistical higher than the portion of genes annotated with this GO-term in the whole genome. We first constructed the GO graph for the yeast ontology in three different categories (biological process, molecular function and cellular component). Then the Hypergeometric Test with FDR multiple hypotheses test correction [12] was applied to calculate and calibrate the *p*-values that indicate the significance of the overlap proportion. In this result, a *p*-value threshold of 0.05 was used. After calculating the GO-term enrichment, we took the minus logarithm for the *p*-values of GO-term enrichment as the log enrichment scores and then summed up the enrichment scores of all statistically significant GO-terms for every TF target gene set respectively for all three GO categories. To take the possible bias from target numbers into consideration, we also calculated the odds ratio enrichment scores by summing the odds ratios of the identified GO terms for each GO category. These steps were done both for the module-inferred functional binding target gene sets and the original binding target genes. Finally, the summary scores between the module-inferred functional target gene set and the original binding targets for each TF were compared.

Compared with the original TF binding dataset, the module-inferred functional binding targets were shown to be better ontology-enriched in all three categories (See Fig. 5 and Additional file 3). First the ontology enrichment tests were performed using the log enrichment scores. For terms categorized in the biological process ontology, 86 out of the 182 TFs (47.2%) showed higher summary enrichment scores in the module-inferred target gene sets while only 29 TFs (15.9%) carried higher summary enrichment scores in the original binding sets (See Fig. 5-a). And for terms categorized in the molecular function ontology, 103 of the 182 TFs (56.6%) demonstrated higher summary enrichment scores in the module-inferred target gene sets and only 22 TFs (12.1%) revealed higher summary enrichment scores in the original target sets (See Fig. 5-b). As for terms categorized in the cellular component ontology, 76 of the 182 (41.8%) module-inferred functional TF target gene sets obtained higher scores while only 18 TFs (9.9%) got higher scores in the original binding dataset (See Fig. 5-c). When the odds ratio scores were considered as the ontology enrichment evaluation metric, the results revealed that 124 (68.1%), 117 (64.3%), 92 (50.5%) of the 182 module-inferred TF target gene sets got larger sums of enrichment odds ratios for the identified GO terms while only 58 (31.9%), 65 (35.7%), 90 (49.5%) of the 182 original TF binding gene sets had higher sums of enrichment odds ratios for the identified GO terms in the biological process ontology, the molecular function ontology and the cellular component ontology respectively. In summary, the module-inferred functional target gene sets are biologically significant by the ontology enrichment test. Thus, the proposed method for mining out TF regulatory modules and inferring functional TF gene targets in yeast is of biological significance in GO term ontology enrichment.

#### TF module-inferred functional targets have better protein interaction prevalence

Proteins usually cooperatively carry out cellular functions through protein-protein interactions. This is usually observed as the form of protein complex in cells [29]. Since genes with similar ontology annotation tend to be regulated by the same TFs, these co-regulated protein-encoding genes are also prone to reveal the prevalence of protein-protein interaction when they involve in similar molecular functions via the form of protein complex. The concept of a protein complex is computationally defined as the definition used in the work of Reimand et al. [14]. A protein complex comprises a core set with protein-encoding genes and the corresponding neighboring gene set. Genes in the target sets are categorized into the core set if they are translated to gene products with physical protein-protein interactions. And genes that are not in the target sets but their gene products are observed to have physical protein-protein interaction with genes in the core set are grouped into the neighboring gene set. Together the core set and the neighboring set form the protein complex of the target gene set. The physical protein-protein interaction information was downloaded from the most recently updated BIOGRID Database [30].

The Hypergeometric Test was used to test the prevalence of protein interaction. A gene set is said to be enriched in prevalence of protein interaction if the proportion of the core gene set to the protein complex is statistically higher than the ratio of the protein complex to the whole genome. Then the FDR multiple hypotheses test correction was applied with a *p*-value threshold of 0.05. Compared with the original TF binding dataset, 68 of the 182 (37.4%) module-inferred functional binding target gene sets were tested to be better enriched in prevalence of protein interaction and only 12 TFs (6.6%) had better protein interaction prevalence enrichment in the original TF binding data (See Fig. 6 and Additional file 4). This demonstrates that the proposed method for mining TF regulatory modules and identifying module-inferred TF functional binding gene targets is biologically significant in the aspect of protein interaction prevalence.

#### TF module-inferred functional targets exhibit better mRNA expression coherence

Genes that cooperatively involved in the same biological process are known to have similar profiles when the mRNA levels expressed by these genes were measured via bio-chips [21]. To fully capture this behavior in different cell conditions, 40 different mRNA expression time series profiles in yeast *Saccharomyces cerevisiae* were collected from ExpressDB [31] and the work of Garten et al. [32]. These 40 different mRNA expression profiles range from the condition of yeast budding sporulation [33], cell cycle gene expression [34, 35], DNA damaging environments [3, 36], yeast metabolism shift [1] and other conditions. We can identify the correlation between the expression profiles of any of the two genes in the target gene sets by calculating their squared Pearson Correlation Coefficients. Then the one-tailed rank-sum test was used to compare the correlation results for the module-inferred functional target gene sets and the original binding target gene sets. The *p*-values in the multiple hypotheses were calibrated by the FDR correction. These three steps were repeated for all TFs considered in this research for the 40 different conditions. A *p*-value threshold of 0.05 was adopted in this test.

The counting summary of conditions with more TFs having higher expression coherence for the 40 different cell conditions is shown in Fig. 7 and Additional file 5 (the details are in Additional file 6). Of the 40 expression conditions, the module-inferred functional targets showed higher number of expression coherent TF target gene sets in 32 expression conditions while in only 7 expression conditions the original binding data got higher expression coherence in the target gene sets. From this result, it is evidenced that the proposed TF regulatory module mining method provides biological significance in practice and the module-inferred functional target genes of TFs can be of real importance in molecular biology.

## Discussions

### Comparison with related works

In yeast, researchers previously have tried to figure out the possible TF sets that might involve in regulatory modules using the concepts of fuzzy set theory and proposed a novel tool called CisMiner [20]. The functional redundancy scores were calculated for the TF sets generated by CisMiner and were compared with the scores of the mined-out modules of the proposed methods in this research. In the work of CisMiner, they considered 102 TFs from the Harbison TF binding dataset and constructed 36 TF regulatory modules for these 102 TFs. Using the Gene ontology (GO) [25] information and the formula of FRS, the functional redundancy scores for the 102 TFs were calculated. Of the 17 TFs that were shown to participate in the 36 CisMiner-proposed TF regulatory modules, they did not have statistically higher functional redundancy scores (mean *FRS*=0.866) than the functional redundancy scores of the rest 85 TFs that were not categorized into any proposed TF regulatory modules (mean *FRS*=0.878). As a comparison, our proposed method can successfully correlate the functional redundancy observed among TFs to the molecular mechanisms of TF regulatory modules (See Fig. 3).

### The proposed method provides experimentally testable TF regulatory module hypotheses

The proposed method in this research tried to elucidate the biological mechanisms behind the functional redundancy observed among TFs by identifying the TF regulatory modules that involve in the co-regulation of gene transcription. These TF regulatory modules provide experimentally testable hypotheses regarding to the gene transcriptional regulation in yeast for biologists. The 238 identified statistically confident TF regulatory modules in yeast were manually checked whether they have been experimentally verified by biologists in the past. These identified TF modules were compared with the YEASTRACT database, a database that manually gathers literature evidence of TF-gene regulation information [9]. A TF module is considered to have literature evidence if all its member TFs are validated to regulate the same gene in experiments performed in one single literature. Overall, 56 (23.5%) of the identified TF regulatory modules were experimentally verified (See Additional file 7).

**Fig. 8:**
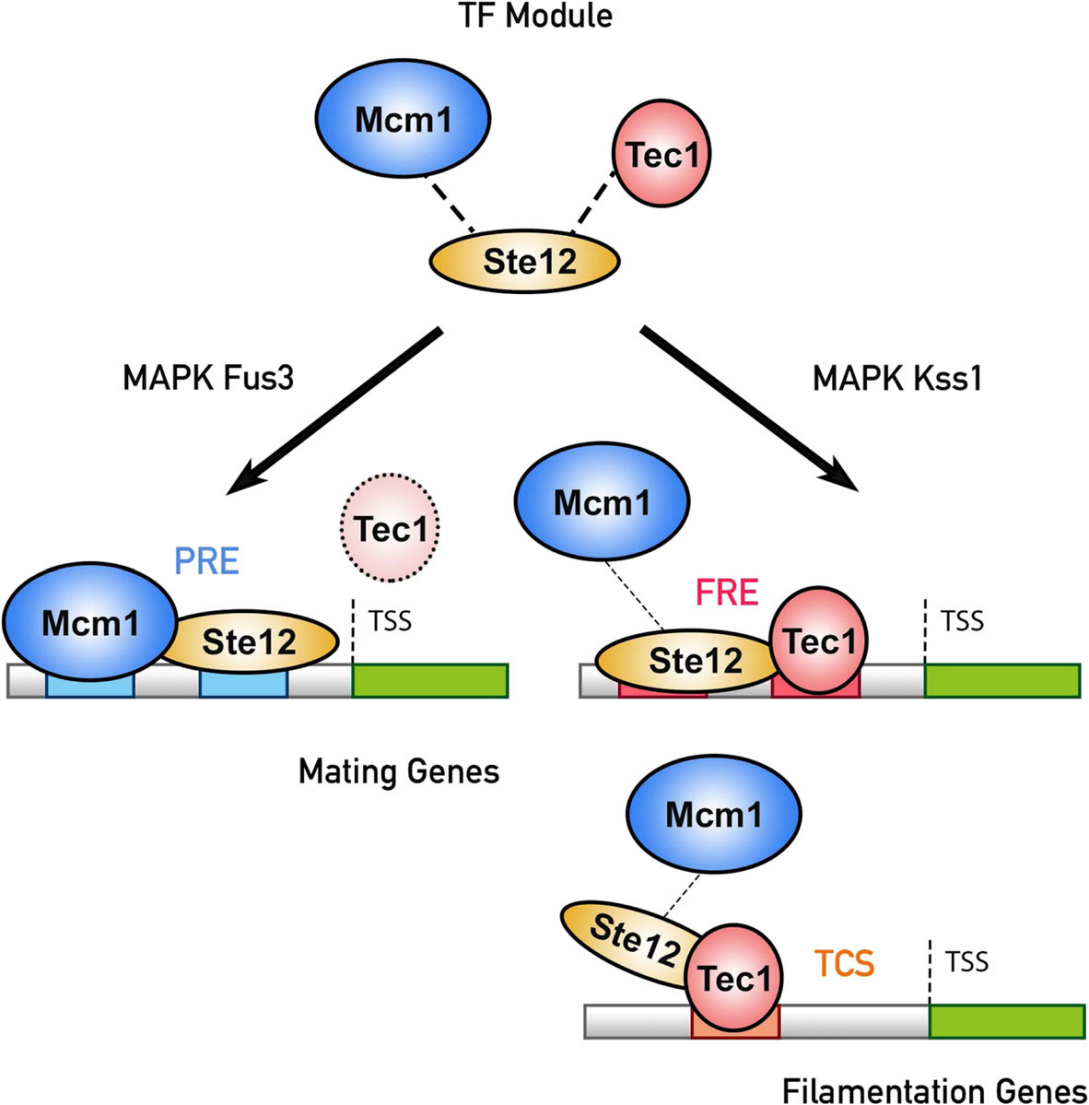
The Ste12 regulatory module is experimentally verified in yeast pheromone response and filamentation. Ste12, Mcm1 and Tec1 are together suggested by the proposed method to form a TF regulatory module. Several studies proved that when the cell is stimulated with mating pheromone, Ste12 undergoes phosphorylation to recognize the PREs in mating genes with Mcm1 and Tec1 is degraded by the proteasome machinery. And it has been verified that when filamentation signals are sensed, Tec1 tethers Ste12 to activate filamentation genes

Here one such verified TF regulatory module is described in detail (See Fig. 8). In yeast, it is known that the transcription factor Ste12, which is regulated by the MAPK cascade, controls two different key developmental programs of pheromone response and filamentous growth [37, 38]. And in the genome-wide identification of Ste12 binding sites adopted from the work of Harbison et al. [4] and Zeitlinger et al. [39], the transcription factor showed binding signals for both the mating genes and the filamentous growth genes. This raised the problem of binding specificity and regulatory program of the TF Ste12. When performing the hypergeometric test to consider the overlap significance between Ste12 and any other TFs in the TF-gene binding dataset, there were 87 TFs showing significant target gene overlaps with Ste12, leading to a combinatorial explosion of possible TF modules to be tested. From this combinatorial candidate explosion, no regulatory program could be easily deduced. Since the original binding dataset contains certain amount of inherent noise signals from the high-throughput experiments, we resorted to both the binding data and the knockout data to get confident functional TF targets. To figure out possible reasons and molecular mechanisms for the problem, the knockout experimental results were collected from the work of Hu et al. [2] and Madhani et al. [40] for subsequent analysis. To get confident functional targets of Ste12 and other TFs, TF binding data and TF knockout data were considered together. When only intersecting the TF ChIP binding data and the TF knockout results, the knockout-inferred functional binding targets of Ste12 consisted of only 53 genes. And considering the overlap significance between Ste12 and any other TFs using the knockout-inferred functional binding target data, no significance overlap with any TF could be found using the functional binding targets identified by simply intersecting the binding data and the knockout data. There is still no hint for the regulatory program and explanatory molecular mechanisms for the binding specificity of Ste12.

To overcome this obstacle, by applying the proposed TF module mining method to genome-wide ChIP experiments and knockout data instead, possible module-inferred functional binding targets were extracted and it is suggested that three TFs Ste12, Mcm1 and Tec1 together form a TF regulatory module. Hence through the proposed method, Ste12 is hypothesized to adjust its function and activate the expression of genes that relate to mating and filamentation respectively under different environmental conditions via this TF module. Several studies demonstrated that Ste12 interacts with Mcm1 physically to regulate the mating genes [41, 42] and Tec1 tethers Ster12 to activate filamentation genes [39, 41, 43]. Ste12 undergoes phosphorylation to recognize the PREs (pheromone-responsive element) in mating genes with Mcm1 when the cell is stimulated with mating pheromone [44]. This process also induces the degradation of Tec1 by the proteasome machinery, thus adjusting the cellular function by mediating the regulatory module [45]. When filamentation signals are sensed, Ste12 is phosphorylated and forms a heterodimer with Tec1 to control filamentation genes with FRE (filamentous responsive element) or TCS (TEA consensus sequence) [46]. These experimental findings coincide with the hypothesized Ste12 regulatory module and provide literature evidence for it. In conclusion, the proposed method can provide valuable and testable molecular mechanism hypotheses.

### Module-inferred functional targets have equal or better biological significance than pure knockout-inferred direct regulatory targets

Using the proposed method to mine out TF regulatory modules in yeast and then utilizing these results to identify the functional binding targets of TFs, 30.8% of the original binding data were obtained to be the module-inferred functional binding targets (See Fig. 3). The biological significance (the ontology enrichment, protein interaction prevalence and mRNA expression coherence) of these module-inferred functional results were further compared with the 4.5% pure knockout-inferred direct regulatory targets obtained by direct intersecting the binding data and the knockout data. As summarized in Table 1, the module-inferred functional targets have equal or better biological significance in all three aspects. In the ontology enrichment analysis using the evaluation metric of log enrichment scores, 128 (70.3%), 109 (59.9%), 150 (82.4%) of the 182 module-inferred TF target gene sets showed equal or better ontology enrichment scores while only 54 (29.7%), 73 (40.1%), 34 (18.7%) of the 182 direct regulatory target gene sets had higher ontology enrichment scores in the biological process ontology, the molecular function ontology and the cellular component ontology respectively. If the odds ratio scores were used as the ontology enrichment evaluation metric, 123 (67.6%), 106 (58.2%), 123 (67.6%) of the 182 module-inferred TF target gene sets showed larger sums of enrichment odds ratios for the identified GO terms while only 59 (32.4%), 76 (41.8%), 59 (32.4%) of the 182 direct regulatory gene sets had higher sums of enrichment odds ratios for the identified GO terms in the biological process ontology, the molecular function ontology and the cellular component ontology respectively. The module-inferred functional TF target gene sets demonstrated better ontology enrichment in both metrics. And 68 (37.4%) out of the 182 module-inferred TF functional binding target gene sets were prevalent in protein interaction while only 13 (7.1%) of the direct regulatory TF target gene sets showed protein interaction prevalence. Finally, in the expression coherence test, the module-inferred functional target gene sets revealed higher expression coherence in 32 conditions while the direct regulatory target gene sets got no higher expression coherence in any of the cellular conditions. In summary, the module-inferred functional target gene sets showed equal or better biological significance than the direct regulatory targets in all three different biological aspects. Thus, the module-inferred functional targets convey equal or higher biological significance as the direct targets and should be of sufficient confidence to be further explored in subsequent research experiments.

**Table 1:** The module-inferred functional binding target gene sets have equal or better biological significance than pure knockout-inferred direct regulatory targets

Biological Aspects		Results
Ontology	Biological	128 (70.3%) TFs showed equal or better log enrichment scores
Enrichment	Process	in module-inferred functional binding target genes while only
		54 (29.7%) TFs showed higher log enrichment scores in the
		direct regulatory data.
	Molecular	109 (59.9%) TFs showed equal or better log enrichment scores
	Function	in module-inferred functional binding target genes while only
		73 (40.1%) TFs showed higher log enrichment scores in the
		direct regulatory data.
	Cellular	150 (82.4%) TFs showed equal or better log enrichment scores
	Component	in module-inferred functional binding target genes while only
		34 (18.7%) TFs showed higher log enrichment scores in the
		direct regulatory data.
Protein Interaction		68 (37.4%) of the module-inferred functional binding gene
Prevalence		sets reveled prevalence of protein interaction while only
		13 (7.1%) direct regulatory gene sets did.
Expression		Module-inferred functional binding genes had higher
Coherence		expression coherence in 32 different conditions while in no
		condition the direct regulatory data got higher expression
		coherence.

### Some other causes may also account for the low overlap between the binding data and the knockout data

Although functional redundancy was shown to help explain the low overlap (4.5%) between the binding data and the knockout data [15], via the proposed method that mined out the molecular mechanisms behind functional redundancy it was shown that functional redundancy only accounts for an extra 26.3% proportion of the original statistically identified TF binding gene targets. The total module-inferred functional binding gene targets (about 31%) were shown to enrich the biological significance of the original binding dataset (See the “Results” section). Hence functional redundancy originated from TF regulatory modules is of high possibility to be part of the reasons for this low overlap. But notice that in this research, it was not deduced that the remaining 69% binding targets to be false positives. It needs to be further investigated to well categorize these remaining 69% binding signals. There may still be some other causes to be further explored. Researchers have also shown that seven properties of genes may be correlated to this low overlap percentage [15]: low expression level, TATA box-less genes, nucleosome occupancy-free regions, low transcriptional plasticity, low number of binding TFs, low number of TFBSs and short average distances of TFBSs to the TSS (transcription start site). These properties might relate to other possible functions of TFs on their target genes and some of them were partially explored. For example, in recent researches it is shown that TFs can help maintain the upstream regions of the TSS in a nucleosome-free state and protect the accessibility against ectopic transcription initiation [47], demonstrating the effect of nucleosome-free regions upstream a TSS on masking the TF-knockout expression change. The TF-gene relationship and the underlying molecular mechanisms of these gene properties require further detailed investigation in future studies.

## Conclusions

Functional redundancy explains part of the reasons of the low overlap between TF binding datasets and the TF knockout datasets. In this research, the concept of TF regulatory module is utilized to propose a novel module mining method for providing biological interpretation on molecular mechanisms of the functional redundancy observed among TFs. It was also demonstrated that the mined-out TF regulatory modules help retrieve functional binding target genes with better biological significance in the protein prevalence study, the ontology enrichment study and the expression coherence study when applied to the original TF binding dataset. Besides that, the proposed method extracted biological significant TF regulatory modules and provided experimental testable hypotheses in the possible modular behavior of transcription regulation in yeast. It is believed that this finding may suggest future research on the modular behavior of the transcription regulation in yeast and will help biologists to further study and understand functions of the *cis*-regulatory modules commonly found in metazo a species.

## Datasets and methods

### TF binding dataset

The genome-wide in vivo cellular TF binding target gene dataset of 203 TFs in baking yeast *Saccharomyces cerevisiae* was downloaded from the work of Harbison et al. [4] and used in this study. They prepared the most comprehensive yeast transcription factor recognition antibodies and used the microarray technology to identity the possible binding gene targets of the known 203 transcription factor in the rich media condition. For interpreting and further analyzing their dataset, a *p*-value of 0.05 as a statistical threshold was adopted for the data. The promoter definition and binding target genes followed the ones used in the study of Harbison et al.

### The TF regulatory module mining algorithm

In this research, a new method to identify TF regulatory modules that reveal the molecular mechanisms for the functional redundancy observed among TFs is proposed. The overall algorithm can be divided into three different stages (See Fig. 1): candidate search stage, module mining stage and noise reduction stage. In the first stage of the proposed algorithm, candidate TF sets were found from the possible combinations of the TFs observed in the TF binding dataset. After that, in the second stage, or the module mining stage, the protein network built from the STRING database was used to extract the most confident but minimal-sized cooperating module for the candidate TF sets. In the noise reduction stage, the random modules that may be formed only by chance or by the noise inherited from the inevitable nature of data integration were eliminated.

#### Candidate search stage

The first stage in the proposed method is to search and select candidate TF sets that may be involved in the same modules. To mine out possible candidate TF sets that may co-regulate a specific target gene, the *k*-means algorithm [22] was used on the expression data since TFs that involve in the same regulatory module tend to have similar cellular mRNA expression profiles. The expression data used in the proposed method consist of 1011 published expression experiments for different cellular conditions collected by Ihmels et al. [21]. In this research, all 1011 conditions were considered and no selection in types of perturbations and treatments was performed. *k*-means is a well-known data mining algorithm that helps learn the group clustering in datasets. And the *k*-means clustering method was applied on the binding TFs of a given gene identified by ChIP experiments to find out possible candidate TF sets. Since the mRNA expression data are of high dimension, special design and pre-processing should be adopted to have *k*-means work in the space formed by the expression dataset. To solve the problem, first the statistical approach PCA (principal component analysis) [48] was used to reduce the high dimensionality of the mRNA expression data while keeping the variation profiles of these 1,011 conditions as a whole. The first three most representative principal components were taken and fed into the *k*-means algorithm in this study. The value of *k* was enumerated from two to the number of binding TFs of a target gene evidenced by the binding dataset. The above steps were repeated for the corresponding binding TF sets of each gene considered in the binding dataset to get all possible candidate TF sets. After this stage, those co-expressed candidate TF sets that may function in a modular manner in regulating specific genes were found.

#### Module mining stage

To mine out the possible molecular mechanisms behind the candidate TF sets, next a protein interaction network based on the data obtained from the STRING database [23] was built. The protein interaction network was modeled as a weighted graph. Nodes in this network represented the proteins deposited in the database and edges were added if there were some literature evidence showing direct protein-protein interaction between the two connected proteins. The weight of an edge was defined to be one minus the confidence level of the evidence. Since the protein interaction data were deposited based on diverse literature evidence, cell conditions and analysis statistical levels, this may contribute some extent of noises to the network. To leverage the information content and noise effect, we mined out the potential TF regulatory modules, which contain the desired candidate TF set and possibly other regulatory proteins, by enforcing the constructed module to have the highest confidence level but with minimal nodes in it. And the cost of an extracted TF regulatory module is defined to be the sum of all the weights of the edges included in the module. Since the extracted potential TF regulatory modules were taken to have the highest literature confidence, which corresponds to the lowest graph path weight sum, or the minimum module cost, these criteria corresponded to choosing the minimal cost Steiner Tree in the constructed network [49].

Finding the minimal cost Steiner Tree of a given network has been proven to be an NP-complete problem [50], which means that it cannot be easily solved under current computation architectures without any constraints [51]. To overcome this obstacle, a modified approximation algorithm based on biological constraints was devised. In cells, the metabolism pathways and modules are usually energy conserved [8, 52]. This means that cells prefer to having fewer participating proteins if available. Based on this assumption, the extracted modules were enforced to have a reasonable weight cost (at most twice the minimum cost [51]) but were constructed to lower the number of participating proteins if possible. The designed method for mining out modules first transformed the network into a metric closure based on the shortest distances between nodes in the interaction network. A metric closure is a complete graph consisting of all the nodes in the protein-protein interaction network. The edge weight between two nodes in the metric closure is set to be the shortest path weight sum between the two nodes in the original network. Then the minimal participating nodes for the given candidate TF set with the moderate weight cost on paths connecting these nodes in this metric closure were found by using the minimum spanning tree algorithm [53]. The accompany proteins in the shortest path information on the minimum spanning tree edges of the metric closure were mapped back to the original protein-protein interaction network and formed the mined-out potential TF regulatory modules. In this way, the proposed method mined out possible TF regulatory modules that provide possible molecular mechanisms for elucidating functional redundancy.

#### Noise reduction stage

Since the integration of data from high-throughput technologies or literature mining is prone to be biased by the intrinsic noises inherited from the experiments, data analysis pipelines and data mining processes, the noised-induced random results in the extracted potential TF regulatory modules were filtered out in the final step of the proposed algorithm. To reduce the effect of noises, it was required that a confident TF regulatory module should have a higher average value of literature confidence for the interactions in this module than the average value of literature confidence for all protein-protein interactions in the whole network. This was tested statistically by using the Mann-Whitney U test [24]. Multiple hypotheses correction was applied to the potential modules mined out for a given gene. The final TF regulatory modules were filtered by a *p*-value threshold of 0.05 in this noise reduction stage.

## Supplementary information

**Additional file 1** The mined-out TF regulatory modules. The TF regulatory modules mined out by the proposed module mining algorithm with their corresponding *p*-values.

**Additional file 2** The module inferred functional binding targets. In this file, the module-inferred functional TF binding target genes for the 182 TFs considered in this research are listed.

**Additional file 3** Ontology enrichment test results. The ontology enrichment tests performed for the module-inferred functional binding targets and the original TF binding dataset in three different ontology categories: biological process, molecular function and cellular component.

**Additional file 4** Protein interaction prevalence test results. The results of protein interaction prevalence enrichment tests for the module-inferred functional targets and the original TF binding data.

**Additional file 5** Expression coherence test summary. In this file, the results of the expression coherence tests for the module-inferred functional targets and the original TF binding dataset in 40 different expression conditions are summarized.

**Additional file 6** Detailed expression coherence test results for the 40 different conditions. In this file, the details of the test results of the expression coherence tests for the module-inferred functional targets and the original TF binding dataset in 40 different expression conditions are archived.

**Additional file 7** Literature evidence of the mined-out TF regulatory modules.

## Data Availability

The datasets supporting the conclusions of this article are included within the article and its additional files.

## Abbreviations

ChIP: Chromatin immunoprecipitation
FDR: False discovery rate
FRE: Filamentous responsive element
FRS: Functional redundancy score
GO: Gene ontology
PRE: Pheromone-responsive element
TCS: TEA consensus sequence
TF: Transcription factor
TFBS: Transcription factor binding sites
TSS: Transcription start site
